# An investigation of dietary intake, nutrition knowledge and hydration status of Gaelic Football players

**DOI:** 10.1007/s00394-020-02341-x

**Published:** 2020-07-30

**Authors:** Conor M. McCrink, Emeir M. McSorley, Kirsty Grant, Andrea M. McNeilly, Pamela J. Magee

**Affiliations:** 1 Nutrition Innovation Centre for Food & Health, University of Ulster, Coleraine, Northern Ireland BT52 1SA UK; 2 Sport and Exercise Sciences Research Institute, University of Ulster, Jordanstown, Newtownabbey, Northern Ireland BT37 0QB UK

**Keywords:** NSKQ, Team sport, Carbohydrate

## Abstract

**Purpose:**

To assess the dietary intake, nutrition knowledge and hydration status of Irish Gaelic footballers.

**Method:**

One hundred and sixty-eight male club/county level Irish Gaelic footballers (median [IQR]; age 23 years [20.0, 27.0]; height 1.79 m [1.74, 1.84]; body mass 78.0 kg [73.5, 84.8]) participated in this cross-sectional study. Dietary intake was assessed using a 4-day semi-quantitative food record, with the application of Goldberg cut-offs to define acceptable reporters (*n*  = 62). Nutrition knowledge was assessed using the validated Nutrition for Sport Knowledge Questionnaire in a sub-group of athletes (*n* = 24), while hydration status was measured using urine specific gravity pre-exercise (USG) in 142 athletes.

**Results:**

Dietary analysis indicated an energy deficit at the group level (485 kcal [IQR 751,6]) (*p* < 0.001), with carbohydrate intakes (3.6 g/kg [IQR 3.0,4.1]) below current guidelines for athletes participating in one hour moderate intensity exercise per day (5–7 g/kg; *p* < 0.001). Average vitamin D (3.8 µg [IQR 1.8, 5.5]) and selenium intakes (54.2 µg [47.2, 76.7]) were significantly below the reference nutrient intakes (*p* < 0.001). A high proportion of individual athletes also had sub-optimal intakes for: vitamin D (95.2%), selenium (72.6%), vitamin A (38.7%), potassium (30.6%), zinc (25.8%), magnesium (19.4%) and calcium (12.9%). Nutrition knowledge was deemed poor (40.2 ± 12.4%), while pre-exercise hydration status (median USG 1.010 [IQR 1.005, 1.017]) was significantly below the cut-off to denote dehydration (1.020; *p* < 0.001).

**Conclusions:**

Our findings suggest that Irish Gaelic footballers have sub-optimal dietary practices and lack nutrition knowledge. Individualised nutrition support may benefit these athletes to meet their nutrition requirements, to support health and performance.

## Introduction

Gaelic football is governed by Ireland’s largest sporting and community organisation, yet there is growing disparity between public and scientific interest in the area [1, 2]. Participation in the sport is two-tier, with universal eligibility for players to represent their local club and through systematic selection, an opportunity to represent their county at the sport’s “elite” level [1]. While amateurism remains a core value at all levels, the competitive expectation and demands placed on athletes are increasingly comparable to that of semi-professional sport [3]. Modern day sport science recognises nutrition as a major facet of an athlete’s ability to perform and recover from exercise [4]. Despite such importance, even professional team sport athletes struggle to meet basic energy and carbohydrate requirements [5].

In elite Gaelic footballers, sub-optimal energy and carbohydrate intakes have also been reported [6, 7]. For an in-season athlete, energy restrictions may have ergolytic and health implications such as reduced recovery, muscle atrophy, fatigue, injury and reduced immune function [8]. Relative energy deficiency in sport is also becoming an increasing concern for athletes with inadequate dietary intakes [9]. The contact nature of Gaelic football also poses risks to musculoskeletal health, with almost a third of the sport’s upper body injuries being fractures [10]. Concerning bone health, the only study to publish micronutrient data in Gaelic footballers indicates inadequate calcium intakes [11], with other findings indicating a high prevalence of vitamin D insufficiency/deficiency [12]. While low energy and unbalanced diets may increase the risk of micronutrient deficiency, sweat may increase losses under conditions of high sweat rates, as may be the case in Gaelic football [13, 14]. Additionally, fluid losses through sweat may increase the risk of dehydration, of which performance decrements are well documented [15]. Although the behaviours that determine nutritional intake are multifactorial, nutrition knowledge may be a key and modifiable element [16]. Previous research indicates that athletes with greater nutrition knowledge are more likely to have greater carbohydrate, fruit and vegetable intakes [17]. Gaelic footballers were previously found to have an inadequate nutrition knowledge [18] using a validated questionnaire. However, as nutrition knowledge is a dynamic process, the use of contemporary and validated tools to mirror the current evidence base is required [16].

At the time of writing [11] has examined the dietary intake of club level Gaelic footballers, however, the limited sample size (*n* = 13) and evolving nutritional practices over a 17-year period merits modern day reassessment. There is also a dearth of sport-specific literature for nutrition knowledge and hydration status in Gaelic football. The primary aim of this study is to quantify the dietary intake of Irish Gaelic footballers and compare these to national [20–23] and sports specific recommendations [4]. Secondary aims are to investigate the nutrition knowledge and hydration status of Gaelic footballers.

## Methods

### Participants

Male Gaelic footballers (*n* = 168) were recruited from Northern Ireland to participate in this cross-sectional epidemiology study. Data collection occurred at two timepoints, using independent clubs during the club in-season period (May 2014–May 2015 and June 2019–July 2019). Participants were screened prior to participation to ensure they met the study inclusion criteria: healthy Gaelic footballers, aged between 18 and 40 years and training at least twice weekly. Anthropometry, dietary intake and hydration status assessment occurred at timepoint one. Anthropometry, dietary intake and nutrition knowledge assessment occurred at timepoint two. This research was conducted in accordance to the Declaration of Helsinki and approval by Ulster University’s Research Ethics Committee (FCBMS-19-017, REC/14/0021). Informed consent was obtained from all participants.

### Anthropometry

Anthropometric measurements were recorded for all participants. Height (cm) was measured to the nearest 0.1 cm using a portable stadiometer (Marsden Leicester Height Measure, Selles Medical, UK). Weight (kg) was recorded using electronic scales (Tanita TBF-310/410) to the nearest 0.1 kg on an empty bladder and in light clothing. Body fat percentage (%) was recorded using bioelectrical impedance (Tanita TBF-310/410) and Body Mass Index (BMI) was calculated using the formula: BMI = weight (kg)/height (m)^2^. All measurements were taken by the trained investigator and in accordance to manufacturer instructions.

### Dietary intake

A total of 91 participants completed a prospective, 4-day semi-quantitative food record. Detailed food, fluid and supplement intake was collected. Quantitative dietary analysis was completed using Nutritics (Nutritics Ltd, Ireland) or Netwisp software (Tinuviel, UK). While no gold standard exists for the assessment of dietary intake, previous research has indicated that 3–7 day recording periods are both pragmatic and reasonably accurate in delineating habitual energy intake in athletes [19]. Average nutrient intake over the 4 days was calculated for the following: energy (kcal day^−1^); macronutrients (g day^−1^ and/or g kg^−1^ day^−1^); and micronutrients (mg day^−1^ or µg day^−1^). Intake data was compared to age and gender specific United Kingdom (UK) national Daily Recommended Values (DRVs) [20–23] and sports nutrition recommendations [4].

### Misreporters

A discrepancy between reported and actual food intake is frequently observed in athletic populations and may lead to systematic error in dietary assessment [24]. To identify misreporters, the revised Goldberg cut-off [25] was applied on an individual and group level for nutritional data in this study:

$$ {\text{EI:BMR}} > {\text{PAL}} \times \exp \left[ {{\text{SD}} _{\hbox{min} } \times \frac{{\left( {S/100} \right)}}{\sqrt n }} \right] $$

$$ S = \sqrt {\frac{{{\text{CV}}_{\text{wEI}}^{2} }}{d} + {\text{CV}}_{\text{wB}}^{2} + {\text{CV}}_{\text{tP}}^{2} } $$

where SD_min_ was − 2 and SD_max_ was + 2 (95% confidence limits), CV_wEI_ intra-subject variation in energy intake = 23%, ${\text{CV}}_{\text{wB}}^{2}$ estimated to measured Basal Metabolic Rate (BMR) precision = 9.8%; ${\text{CV}}_{\text{tP}}^{2}$, inter-subject physical activity level (PAL) variation = 15%; *d* days of dietary assessment = 4 [25]. BMR was estimated using predictive equations [26]. A PAL value of 1.6 (moderate activity) was selected for study participants [27]. At the group level, calculated cut off points were < 1.53 and > 1.61 for under-reporters (URs) and over-reporters (ORs), respectively. At an individual level, a cut off of < 1.04 and > 2.40 was calculated to define URs and ORs, respectively. Only acceptable reporters (ARs) were included in statistical analysis. A post hoc sensitivity analysis that included misreporters was used to determine the robustness of findings [27].

### Sports nutrition knowledge

The recently validated eighty-nine question Nutrition for Sport Knowledge Questionnaire (NSKQ) [28] was disseminated to a sub-group of Gaelic footballers (*n* = 24) in hardcopy format following a training session. The NSKQ is separated into six subsections (weight management, macronutrients, micronutrients, sports nutrition, supplements and alcohol). Demographic information was also collected. Scoring followed the criteria of one point for a correct response, whereas an incorrect or “unsure” response received zero. As the NSKQ was developed in Australia, demographic questions were tailored to suit the Irish population. Scores (%) for nutrition knowledge were interpreted as follows: 0–49 (poor), 50–64 (average), 65–74 (good), ± 75 (excellent), based on results previously published [29].

### Hydration status

Urine Specific Gravity (USG) was used to assess the hydration status of athletes (*n* = 142) using a digital refractometer (Atago PAL-10S, Cole-Parmer UK), to an accuracy of ± 0.001. Refractometer USG is both reproducible and accurate for hydration status assessment [18]. Following the pre-exercise collection of urine (~ 10 ml), samples were analysed within one hour. The refractometer was calibrated using distilled water prior to urine analysis, with recalibration every three samples. A USG cut off point of > 1.020 was used to define dehydration [15].

### Statistical analysis

Statistical analysis was completed using Statistical Package for the Social Sciences (SPSS *v25*, IBM, Chicago, IL, USA). The distribution of data was assessed using Shapiro–Wilk and Q–Q plots to ensure appropriate testing and reporting of data. Statistical significance was set at *p* < 0.05 and all tests were two-tailed. Median (interquartile range) was used for distributions that violated the assumption of normality, whereas mean ± SD was used to report normal data. A Mann–Whitney *U* test was used to compare demographic information between URs and ARs. A Wilcoxon signed-rank test was conducted to compare macronutrient/micronutrient intakes with the DRVs or sports nutrition recommendations, in addition to comparison of USG to the dehydration cut-off. A one-sample *t* test was used to compare total and sub-section scores to NSKQ scoring criteria.

## Results

### Demographics

Participant demographics are presented in Table 1. At the group level, Gaelic footballers were categorised as URs. At the individual level, no participant was categorised as an OR, however, 29 were categorised as URs and 62 as ARs. URs had a significantly higher bodyfat percentage, weight and BMR (Table 1).

**Table 1:** Demographic characteristics of Gaelic footballers

Characteristic	Total cohort (*n* = 168)	Under-reporters (*n* = 29)	Acceptable reporters (*n* = 62)	*P*-value
Age (years)	23.0^$^ (20.0, 27.0)	22.0 (19.0, 27.0)	23.0 (20.8, 27.0)	0.178
Weight (kg)	78.0 (73.5, 84.8)	82.0* (76,2, 89.5)	77.7 (73.5, 84.5)	0.025
Height (m)	1.8 (1.7, 1.8)	1.8* (1.8, 1.8)	1.8 (1.7, 1.8)	0.045
BMI (kg/m^2^)	24.6 (23.3, 25.8)	25.2 (23.4, 26.7)	24.8 (23.5, 25.8)	0.299
Body fat (%)	14.8 (12.1, 19.7)	18.2* (13.1, 22.7)	14.2 (11.7, 19.0)	0.010
BMR (kcal·day^−1^)	1786.6 (1709.6, 1895.4)	1848.6* (1784.5, 1978.3)	1788.4 (1702.4, 1888.2)	0.022
EI:BMR ratio^ǂ^	1.2 (0.9, 1.4)	0.8* (0.7, 0.9)	1.3 (1.2, 1.6)	< 0.001

*kg* kilograms, *%* percentage, *BMR* basal metabolic rate, *EI:BMR* energy intake to BMR ratio, *kcal* *day*^*−1*^ energy per day

^$^Values presented as median (interquartile range)

*Significant difference relative to acceptable reporters as determined by Mann–Whitney *U* test (*p* < 0.05)

^ǂ^Total cohort, *n* = 91

### Energy and macronutrient intake

Average energy and macronutrient intake for Gaelic footballers defined as ARs (*n* = 62) was compared to national DRVs/sports nutrition recommendations (Table 2). Energy intake was significantly lower than requirements, with 75.8% of athletes in an energy deficit (485 kcal [IQR 751,6]) compared to individual estimated requirements. Total intake of protein, fat (% energy intake) and alcohol met recommendations. Only 4.8% of athletes met carbohydrate recommendations to reflect the demands of 1-h moderate intensity exercise per day [4]. Similarly, average carbohydrate intake was also below the DRV. Fibre, monounsaturated and polyunsaturated fats were significantly below recommendations, whereas the intake of free sugar and saturated fat were significantly above recommendations. Comparisons remained robust following sensitivity analysis.

**Table 2:** Average energy and macronutrient intake of Gaelic footballers (*n* = 62)

Nutrient	Intake (median [IQR])	National DRV targets/SNRs	Athletes meeting DRV or SNR target/range	*P*-Value*
			*N* (%)^†^	
Energy				
Kcal day^−1^	2496.2 (2162.2, 2719.1)	2861^e^	15 (24.1)	< 0.001
Protein				
Total, g	114.2 (96.4, 125.2)	55.5^a^	62 (100)	< 0.001
g kg^−1^ day^−1^	1.4 (1.2, 1.7)	1.2–2^d^	43 (69.3)	< 0.001
% EI	18.0 (16.4, 20.8)	15^a^	55 (88.7)	< 0.001
Carbohydrates				
Total, g	290.7 (234.1, 319.2)	NA	NA	NA
g kg^−1^ day^−1^	3.6 (3.0, 4.1)	5–7^d^	3 (4.8)	< 0.001
% EI	46.4 (41.2, 49.4)	≥ 50^ab^	14 (22.6)	< 0.001
Free sugar, % EI	8.8 (4.9, 12.3)	≤ 5^b^	15 (24.2)	< 0.001
Fibre, g	21.5 (18.5, 25.8)	≥ 30^b^	6 (9.7)	< 0.001
Fat				
Total, g	87.0 (75.5, 97.3)	NA	NA	NA
g kg^−1^ day^−1^	1.1 (1.0, 1.3)	NA	NA	NA
% EI	32.2 (28.5, 36.2)	20–35^d^	43 (69.4)	< 0.001
SFA, % EI	11.7 (10.0, 13.1)	≤ 10^c^	25 (40.3)	0.07
MUFA, % EI	11.3 (9.6, 13.0)	≥ 13^a^	16 (25.8)	< 0.001
PUFA, % EI	4.5 (3.4, 5.5)	6.5–10^ac^	7 (11.3)	< 0.001
Alcohol				
Total, % EI	0.0 (0.0, 9.1)	< 5^a^	42 (67.7)	0.08

*IQR* interquartile range, *DRV* daily recommended value, *EI* energy intake, *SNR* sports nutrition recommendations, *BMR* basal metabolic rate, *SFA* saturated fatty acids, *MUFA* monounsaturated fatty acids, *PUFA* polyunsaturated fatty acids, *kcal* Kcal day^−1^, energy per day, *g* *kg*^*−1*^ *day*^*−1*^ grams per kilogram per day, *NA* not applicable, *n* participants

*P-value determined by one-sample Wilcoxon signed-rank test. Intake compared to DRV/SNR. Data compared to lower (protein, PUFA, fat g kg^−1^ day^−1^) or upper range (fat %EI), where appropriate

^†^Protein g kg^−1^ day^−1^,fat %EI (between range used) and energy intake compared to individual requirements

^a^COMA [20]

^b^SACN [23]

^c^SACN [22]

^d^Thomas et al. [4]

^e^BMR × PAL (1.6)

### Micronutrient intake

Average micronutrient intake for ARs was compared to national DRVs (Table 3). For the total cohort, micronutrient intakes were significantly above the Reference Nutrient Intake (RNI) (*p* < 0.005), with the exception of selenium and vitamin D which were below the RNI (*p* < 0.001). Comparison to recommendations remained robust following sensitivity analysis. On an individual level, 3.2% of athletes were below the Lower Reference Nutrient Intake (LRNI) for vitamin A and 12.9% were below the LRNI for selenium. In comparison to the RNI, a high proportion of athletes had sub-optimal intakes for: vitamin D (95.2%), selenium (72.6%), vitamin A (38.7%), potassium (30.6%), zinc (25.8%), magnesium (19.4%) and calcium (12.9%).

**Table 3:** Average micronutrient intakes of Gaelic footballers with comparison to DRVs (*n* = 62)

Micronutrient	Intake (median [IQR])*	DRVs^†^		
		LRNI (% meeting)	EAR (% meeting)	RNI (% meeting)
Vitamins				
Vitamin A, µg	859.5 (578.5, 1165.9)	300 (96.8)	500 (83.9)	700 (61.3)
Vitamin D, µg	3.8 (1.8, 5.5)	NA	NA	10 (4.8)
Vitamin E, mg	10.0 (6.9, 12.6)	NA	NA	4 (100)
Thiamin (B1), mg	2.3 (1.8, 2.7)	0.6 (100)	0.8 (100)	1.0 (100)
Riboflavin (B2), mg	2.3 (2.0, 3.1)	0.8 (100)	1.0 (96.8)	1.3 (93.5)
Niacin (B3), mg	58.8 (46.4, 70.0)	11.2 (100)	14.0 (100)	16.8 (100)
Folate (B9), µg	345.4 (279.8, 425.4)	100 (100)	150 (100)	200 (98.4)
Vitamin B12, µg	6.2 (5.2, 9.4)	1.0 (100)	1.25 (100)	1.5 (100)
Vitamin C, mg	91.3 (55.5, 130.9)	10 (100)	25 (98.4)	40 (95.2)
Minerals				
Sodium, mg	2793.7 (2338.1, 3294.7)	575 (100)	NA	1600 (96.8)
Potassium, mg	3796.5 (3386.2, 4408.0)	2000 (100)	NA	3500 (69.4)
Magnesium, mg	354.5 (312.1, 426.7)	190 (100)	250 (95.2)	300 (80.6)
Calcium, mg	1080.9 (812.4, 1420.6)	400 (100)	525 (96.8)	700 (87.1)
Iron, mg	14.1 (11.6, 17.5)	4.7 (100)	6.7 (100)	8.7 (96.8)
Zinc, mg	11.6 (9.3, 15.6)	5.5 (100)	7.3 (93.5)	9.5 (74.2)
Selenium, µg	54.2 (47.2, 76.7)	40 (87.1)	NA	75 (27.4)

*IQR* interquartile range, *DRV* daily recommended value, *LRNI* lower reference nutrient intake, *EAR* estimated average requirement, *RNI* reference nutrient intake, *NA* not available, *n* number of participants

*Significant difference compared to RNI as determined by one-sample Wilcoxon signed-rank test (*p* < 0.005)

^†^DRVs from COMA [20], SACN [21]

### Nutrition knowledge

**Fig. 1:**
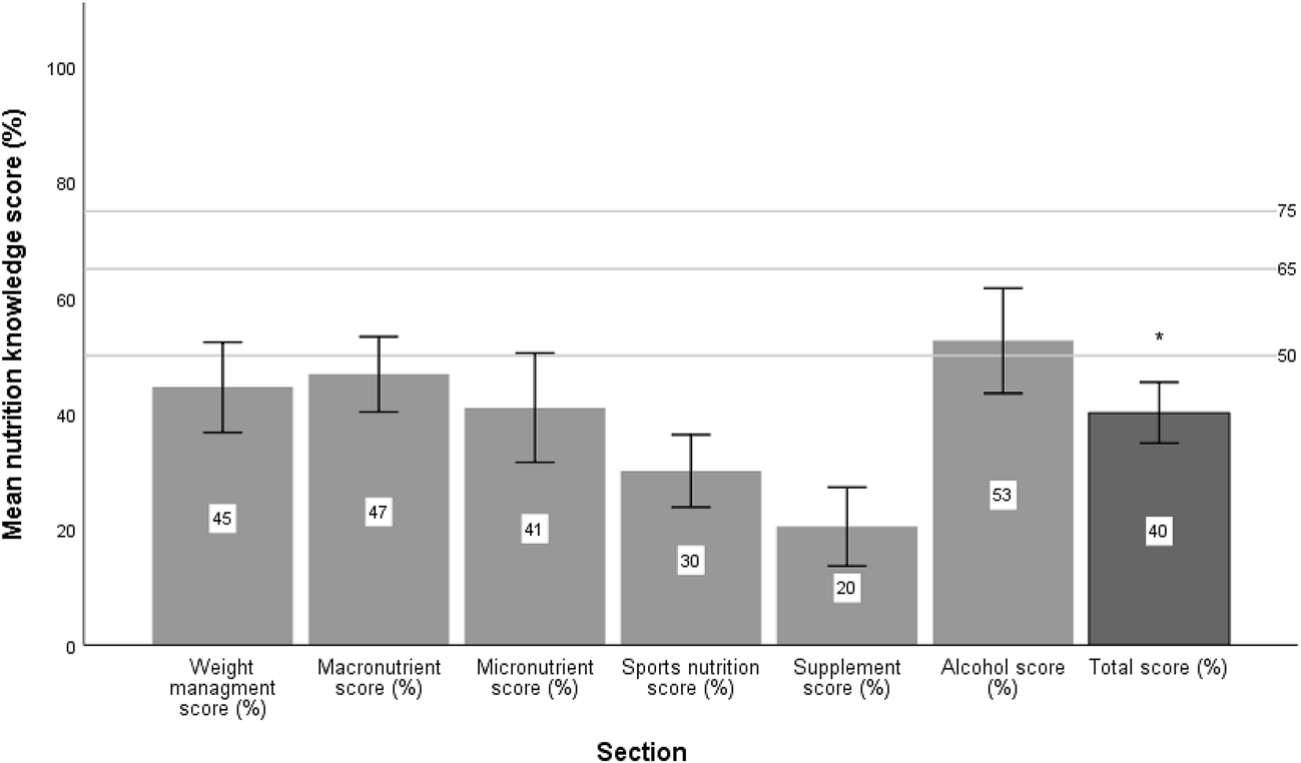
Results from the NSKQ for Gaelic footballers (*n* = 24). Values are mean with 95% confidence intervals. Horizontal lines denote criterions for performance in the questionnaire: 0–49 (poor), 50–64 (average), 65–74 (good), ± 75 (excellent). *Total score significantly lower than criterion for average nutrition knowledge as determined by one-sample t-test (*p* = 0.001)

Twenty-four athletes completed the NSKQ (Fig. 1). Mean total score for the sample (40.2 ± 12.4%) fell within the “poor” nutrition knowledge category (*p* = 0.001). Many (79.2%) participants had “poor” nutrition knowledge and the remainder were categorised with “average” nutrition knowledge. Sports nutrition (30.1 ± 14.9%) and supplement (20.5 ± 16.1%) sub-sections were significantly lower than the criterion for “average” nutrition knowledge (*p* < 0.001), whereas no significant difference was noted for: macronutrients (46.8 ± 14.5%), weight management (44.6 ± 18.4%), micronutrients (41.0 ± 22.3%) and alcohol (52.6 ± 21.5%) (*p* > 0.05). All sub-sections were below “good” and “excellent” nutrition knowledge (*p* < 0.05). The majority (87.5%) of participants responded that they would like clubs to provide access to Nutritionists/Dietitians, with the remaining (12.5%) desiring nutrition information only. The most useful nutritional support was deemed to be individual consultations (41.7%) and least useful being group presentations (4.2%).

### Hydration status

**Fig. 2:**
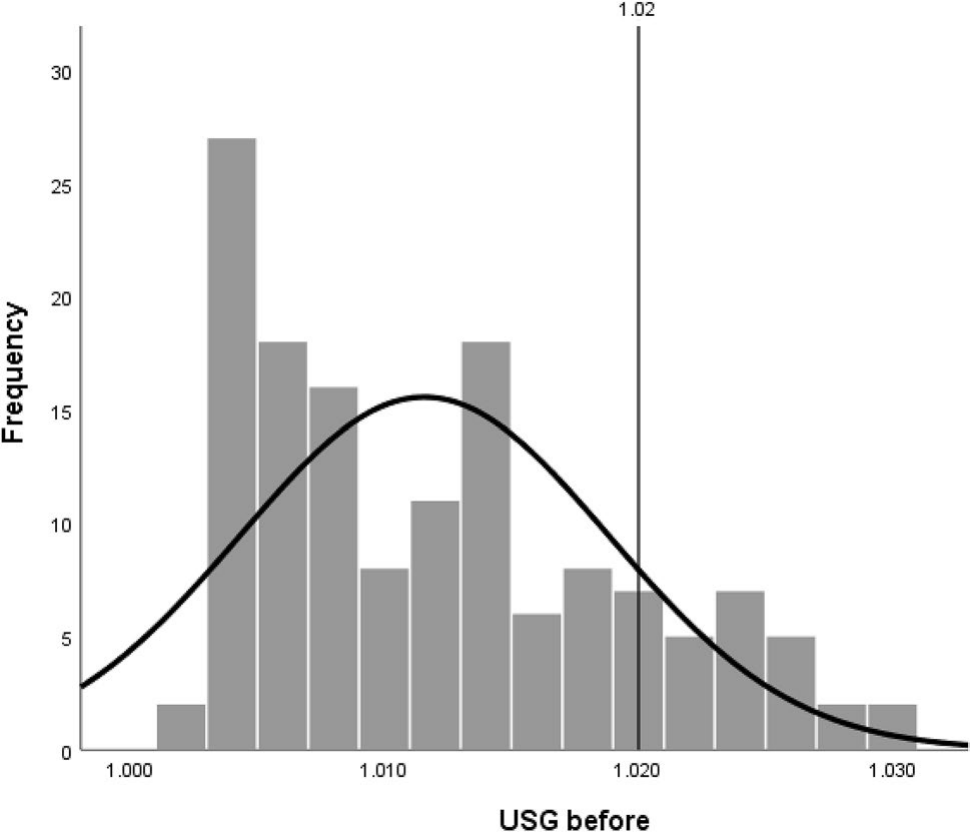
Distribution of median urine specific gravity for Gaelic footballers (*n* = 142) before training. Vertical line at 1.020 depicts cut-off point to indicate dehydration, with 19% of participants exceeding this threshold. Median USG significantly differed from the dehydration cut-off point as determined by one-sample Wilcoxon signed rank test (*p* < 0.001)

Pre-exercise hydration status of Gaelic footballers (*n* = 142) is displayed in Fig. 2; 19% of athletes were dehydrated prior to exercise (USG > 1.020). Median USG (1.010 [IQR 1.005, 1.017]) was significantly below the cut-off point to signify dehydration (*p* < 0.001).

## Discussion

To our knowledge, this is the first study to investigate both macronutrient and micronutrient intakes, in addition to the nutrition knowledge and hydration status of Gaelic footballers. Key findings indicate inadequate dietary intakes of energy, carbohydrates, vitamin D and selenium. A high proportion of athletes were also below the RNI for vitamin A, potassium, zinc, magnesium and calcium. Although only assessed in a sub-group of the footballers, we can conclude that nutrition knowledge was poor, while the majority of athletes commenced training euhydrated.

There was a high prevalence of under-reporting within this cohort, with a significant relationship between higher bodyfat percentage and under-reporting noted. Similar characteristics of under-reporters have been demonstrated previously [30], which may be a consideration for practitioners and researchers working with Gaelic footballers. Despite the exclusion of under-reporters, these findings indicate that energy intake was inadequate to meet training demands, with an average daily energy deficit of 485 kcal (IQR 751,6). Insufficient carbohydrate intake appears largely responsible for this observation (Table 1). An intentional restriction of carbohydrate may be possible considering lower levels of bodyfat observed in this cohort (14.8% [IQR 12.1, 19.7]) relative to other club level Gaelic footballers (18.3 ± 3%) [11]. Energy restriction by method of carbohydrate reduction has been documented previously in athletic cohorts to reach perceived body composition goals [31]. A greater understanding of the rationale for sub-optimal carbohydrate intakes is needed and may indicate the need for education and knowledge, a poor domain when measured (Fig. 1).

Inadequate carbohydrate intake is common among Gaelic footballers [6, 7, 11] and team sports such as soccer, rugby and Australian rules football [5]. Concerning in-season performance, carbohydrates likely form a key metabolic substrate considering the sport’s intermittent activity profile [32]. Failure to maintain carbohydrate availability via glycogen or exogenous sources may have ergolytic implications including fatigue, impaired recovery and increased perception of effort [4]. Recently popularised strategies such as “training low” may foster dissonance for athletes understanding the significance of these recommendations. The performance benefits of such strategies lack consensus and misuse may have ergolytic potential [33]. It should also be noted, however, that the actual carbohydrate requirements of Gaelic football can only remain speculative in the absence of research investigating glycogen utilisation of the sport. As current sports nutrition guidelines are largely derived from endurance sports data, their application to team sports may be questioned [34]. While a consideration for future research, present findings remain concerning with the majority of players failing to meet general public health guidelines, which likely form an underestimation for athletic cohorts. Further concerns of lower carbohydrate diets may be reduced dietary fibre intakes, as demonstrated in this cohort (Table 2). Emerging evidence suggests that dietary fibre is associated with a reduced risk of colorectal cancer, cardiovascular disease and type II diabetes [35]. In the interest of health, Gaelic footballers should aim to increase fibre intake outside the workout period to mitigate the potential caveat of gastrointestinal distress during exercise [4].

The plurality of research into team sport athletes suggests that protein intake typically meets or exceeds recommendations [5], including Gaelic football specifically [6, 7]. Considering similar findings of the present study, perhaps future research should focus on quality and distribution of intakes as current understanding of muscle protein synthesis develops [36]. Fat intake was also within recommendations, however, the type of fat consumed also merits consideration. Consistent with UK population data, saturated fat intakes in this present study were elevated [22]. Due to adverse associations with lipid biochemistry and the incidence of cardiovascular events, it may be advisable for Gaelic footballers to reduce saturated fat and of importance, replace with unsaturated sources within the energy budget allocated to fat [22]. While further research in the area is required, preliminary evidence also suggests specific benefit of omega-3 polyunsaturated fat, in relation to factors relevant to athletic cohorts including, muscle recovery, muscle protein synthesis and immune function [37].

Dehydration inducing hypovolemia may increase core temperature, glycogen utilisation and cardiovascular strain [15]. As such, commencing exercise euhydrated and minimising fluid losses during activity is recommended [4]. The majority of Gaelic footballers within this study commenced exercise euhydrated, consistent with previous sport specific findings [14, 18]. The risk of hypohydration during sporting activity, however, remains equivocal from these studies. Newell et al. [14] reported a high sweat rate (1.4 litres/hour), whereby many (60%) athletes developed dehydration post-exercise, however, Magee et al. [18] concluded the majority of female Gaelic footballers remained euhydrated post-exercise. This may be explained by lower sweat rates in female athletes [15] or the reliance on percentage bodyweight loss as criterion for dehydration post-exercise by Newell et al. [14]. A measure of dehydration using percentage weight loss may be an inaccurate criterion for those already commencing training in a dehydrated state [18]. Further research with male Gaelic footballers using validated measures both pre and post exercise appear warranted. Gaelic footballers should be educated on the importance of commencing exercise euhydrated and the factors that determine sweat rate. It may also be prudent for Gaelic footballers to drink according to an individualised fluid plan to minimise the risk of dehydration with associated health and performance implications [1]. Breaks between play and half time periods should be utilised to achieve this fluid plan.

Previous research has recommended the study of micronutrient intakes of Gaelic footballers [1]. In this present study, both selenium and vitamin D intakes fell below the RNI. While selenium intake of the adult UK population is lower (48 µg day^−1^) than current findings, this was not deemed to have adverse health outcomes when investigated [38]. Inadequate intakes of vitamin D, however, can cause pathophysiology of the musculoskeletal system, leading to reduced bone mineral density, impaired muscle strength and stress fractures [21]. It should be noted though that cutaneous synthesis from sunlight may have occurred during the summer months, which is not considered in dietary analysis. Although risk remains present, as with other RNIs, biochemical analysis is required to diagnose clinical deficiency [39]. Nevertheless, a proportion of the athletes (12.9%) had inadequate calcium intakes, perhaps raising bone health concerns during winter months for Gaelic footballers, where serum vitamin D levels were previously inadequate [12]. Physical activity may also inherently increase the risk of micronutrient deficiency in athletic cohorts through increased requirements relating to various factors such as sweat and metabolism. Current consensus, however, suggests that the correlation between energy intake and micronutrients may meet this additional demand if an athlete’s intake is balanced and high in carbohydrates [40]. Unfortunately, present findings demonstrate this assumption does not appear accurate in Gaelic footballers. Practitioners should aim to educate players on nutrient dense food options to support their individual needs. Vitamin D supplementation may also be prudent, especially during the winter months [21].

The apparent poor nutrition knowledge of the Gaelic footballers within this study may have influenced dietary intake. Nutrition knowledge (40.2 ± 12.4%) was lower than previous findings in Irish athletes by Walsh et al. [41] (59.6 ± 12.8%) and Magee et al. [18] (53% [IQR 46.0, 59.8]). These comparisons, however, are limited due to heterogeneity of sampling tools. Recent findings in Australian athletes (*n* = 154) using the NSKQ indicate marginally better nutrition knowledge (48.2 ± 12.1%), although still classified as poor [42]. Poor nutrition knowledge is, therefore, a prevalent issue among athletes and requires corrective action. Interestingly, athletes of the current study indicated that group presentations were the least useful form of obtaining nutrition information, with individual consultations being favoured, similar to reports from Australian athletes [42, 43]. With the majority of athletes (87.5%) interested in services from a Registered Dietitian or Nutritionist, this should be considered by Gaelic football clubs to promote player welfare.

The present study has some limitations. The use of food records in dietary assessment may be prone to inherent reporting errors, typically in the direction of under-reporting [31]. While acknowledged by the use of Goldberg cut-offs, errors are also pertained with this method, most notably, the assumption of PAL. In the absence of objective measures of energy expenditure, a conservative PAL value was selected with knowledge of this cohort’s training demands [1]. It remains plausible that athletes were erroneously classified as under-reporters due to undereating, rather than under-recording [31], nevertheless, the use of sensitivity analysis indicates robust findings. The NSKQ has also been subject to recent modification following feedback since development [44]. Fourteen items were altered following this update, which may have affected present findings. Nutrition knowledge was also only assessed on a small subset of participants from one club, therefore, extrapolation should be made with caution. Provided the small sample size, correlation between dietary intake and nutrition knowledge was not made, which may have affirmed the relationship between knowledge and intake. Association between other parameters such as hydration and nutrition knowledge was also limited as these were assessed in different groups of players. It should be noted, however, that the cross-sectional study design was always limited to correlation and not causation. Hydration status was measured pre-training for only one cohort and environmental factors (e.g. temperature) were not recorded. This may have provided useful context to elaborate on findings. Future research should aim to address these limitations. The inclusion of female Gaelic footballers and investigations of energy availability in Gaelic footballers are also warranted.

In conclusion, these findings indicate that male Gaelic footballers exhibit sub-optimal energy, carbohydrate, vitamin D and selenium intakes relative to current recommendations. The nutrition knowledge of Gaelic footballers is also poor, while the majority of players enter training euhydrated. Adequate nutritional intake is of paramount interest to promote optimal performance and safeguard athletes from the health related implications of nutrient related deficiencies. As a modifiable and lacking element of dietary behaviour, improvements in nutrition knowledge are required. Practical strategies should be developed by clubs, with consideration of individualised player support by qualified professionals, if possible.
